# Contrasting mechanistic susceptibilities of hematopoietic and endothelial stem-progenitor cells in respective pathogeneses of HIV-1 and SARS-CoV-2 infections

**DOI:** 10.3389/fcell.2023.1296986

**Published:** 2023-12-06

**Authors:** Prasad S. Koka, Bharathi Ramdass

**Affiliations:** Biomedical Research Institute of Southern California, Oceanside, CA, United States

**Keywords:** stem-progenitor cells, hemangioblasts, virus-cell interactions, direct and indirect target cells, cellular dysfunction and dysregulation, pathogenesis, post-transcriptional regulation, microRNA

## Abstract

The multitude of cellular types can be expected to behave differently when receiving invading pathogens such as mammalian viruses. The nature-dictated causes for such intrinsic cellular diversity become the criteria for the emergence of specific virus-receptor interactions on that particular host cellular surface, in order to accommodate contact with various other living entities whether desirable to the host or not. At present, we are presented with an example of two contrasting behaviours wherein the well-known HIV-1 and the more recently emergent SARS-CoV-2 cause adverse consequences to the differentiation and functions of progenitor stem cells. These include the two different downstream multipotent CD34^+^ hematopoietic (HSPC) and CD133^+^ endothelial (ESPC) stem-progenitor cells of their common pluripotent hemangioblast precursors. The two viruses target the respective endothelial and hematopoietic stem-progenitor cells to thrive upon the relevant host cellular surrounded stromal microenvironments by adopting reciprocally-driven mechanistic routes, which incidentally cause pathogenesis either directly of ESPC (SARS-CoV-2), or indirectly of HSPC (HIV-1). HIV-1 utilizes the CD4^+^ T-lymphocyte receptor thereby advancing pathogenesis indirectly to the CD34^+^ HSPC. SARS-CoV-2 directly targets the CD133^+^ ESPC via ACE2 receptor causing cytokine storms of the CD4^+^ T-lymphocytes. In this manner, these two viruses cause and extend their damage to the other cellular sub/types coexisting in the host cellular microenvironments. The infected individuals require clinical interventions that are efficacious to prevent cellular dysfunction and ultimate cell depletion or death. We infer from these viruses mediated pathogeneses mechanisms a potential common origin of microRNA molecular therapies to address cellular dysfunctions and prevent cell loss.

## Introduction

In general, the mammalian viruses target specific receptors of the host eukaryotic cells. More specifically, these human cell infection-tropic viruses include the cellular primary receptors CD4 (T-helper-cell marker antigen) utilized by HIV-1 (Withers-Ward et al., 1997; Yunis et al., 2007) and angiotensin-converting enzyme-2 (ACE2) utilized by SARS-CoV-2 (Gheblaw et al., 2020; Kotani et al., 2022). As expected, these two different viruses target and initially enter the human body through these defined receptors and co-receptors CCR3, CCR5, or CXCR4 for HIV-1 (Zhang et al., 2010)**,** and TMPRSS2, NRP1, or CD147 for SARS-CoV-2 (Cantuti-Castelvetri et al., 2020; Daly et al., 2020; Hoffmann et al., 2020; Wang et al., 2020; Gudowska-Sawczuk and Mroczko, 2021; Shapira et al., 2022)**,** expressed on cells of different virus-tissue-tropism, resulting in varied pathological progression of infectious diseases threatening the very survival of the infected humans. One of the main and major features of these two viruses is that whereas HIV/AIDS is currently known to be responsive to multidrug antiretroviral (ART) treatment and generally not cause immediate human death, SARS-CoV-2/COVID-19 on the other hand exhibited rapid lethality in certain population demographics, particularly those experiencing comorbidities. HIV/AIDS has been brought under control even though the extremely high and rapid replicative mutation rates of HIV-1 have yet remained elusive for vaccination. However, COVID-19 has been generally contained although after millions of human deaths worldwide, with rapid development of multiple efficacious vaccines from across the world. Nonetheless, this does not imply that any newly emerging SARS-CoV-2 variants may not be able to evade containment by the currently available vaccines. The development of new vaccines will need to keep pace and be current with the newly emerging SARS-CoV-2 replicative mutations and thus this virus’ variants. In this *Perspective* article, we discuss the modalities and salient phenotype-driven mechanistic-contrast features in the onset and progress of pathogenesis of these two viruses upon infection. We hypothesize that specific and yet-to-be-identified or characterized microRNA molecules may be involved in the SARS-CoV-2 mediated pathogenesis of this virus’ infection-resistant (primary ACE2 receptor-lacking) secondary T-lymphocyte cellular targets (Hikmet et al., 2020). SARS-CoV-2 primary infection via ACE2 triggers massive secondary potentially unabated cytokine storms of T-lymphocytes released into the systemic circulation of clinically vulnerable individuals (Fajgenbaum and June 2020; Liu et al., 2021). In this context, we describe and postulate that potential specific microRNAs may be involved in the CD133^+^ endothelial stem-progenitor cell-mediated T-lymphocyte dysfunction in SARS-CoV-2 infection (COVID-19), showing a certain similarity to the incidence of miRNA dysregulation mechanisms of pathogenesis that occur in HIV-1 infection (Padmanabhan et al., 2020). Thus, SARS-CoV-2 may confer via ACE2^+^CD133^+^ ESPC (Koka et al., 2020)**,** a contrasting reciprocal involvement of miRNA in the onset of cytokine storm in T-lymphocytes, and the mechanistic characterization of such may in the future pave the way for novel treatments for COVID-19.

### Target host cellular receptors of HIV-1 and SARS-CoV-2 and role reversal

The human host target molecular receptors of the two viruses HIV-1 and SARS-CoV-2 dictate the types or nature of human host cells for each virus entry to initiate primary infection. CD4 expressed on T-lymphocytes is the natural primary receptor with CCR3, CCR5, or CXCR4 (Zhang et al., 2010) secondary co-receptors for HIV infection. Angiotensin-converting enzyme-2 (ACE2) is the natural primary receptor for SARS-CoV-2 infection (Gheblaw et al., 2020; Kotani et al., 2022) together with Transmembrane protease serine-2 (TMPRSS2), Neuropilin-1 (NRP1), or Basigin (CD147) serving as the secondary co-receptors (Cantuti-Castelvetri et al., 2020; Daly et al., 2020; Hoffmann et al., 2020; Wang et al., 2020; Gudowska-Sawczuk and Mroczko, 2021; Shapira et al., 2022). Hence, the required CD4 and ACE2 primary receptors for HIV-1 and SARS-CoV-2 entry into CD4^+^ T-lymphocytes and CD133^+^ ESPC respectively with the targeted cells not only become pathogenically susceptible to the particular virus but also indirectly affect the CD34^+^ HSPC (in HIV/AIDS) and CD4^+^/CD19^+^ T/B-lymphocytes (in SARS-CoV-2/COVID-19) respectively. Thus, these two viruses exhibit a common pluripotent hemangioblast precursor (CD34^+^CD133^+^) differentiated multipotent stem-progenitor cell (HSPC/ESPC) vis-à-vis T-lymphocyte reciprocity to cause respective secondary target cell influence resulting in cellular dysfunction, depletion, or death.

### Indirect and direct involvement of human stem-progenitor cells

SARS-CoV-2 directly targets the precursors which are the pluripotent hemangioblasts that possess lineage commitment into the dual multipotent lineages of CD34^+^ hematopoietic stem-progenitor cells (HSPC) and CD133^+^ endothelial stem-progenitor cells (ESPC) (Sidney et al., 2014; Hassanpour et al., 2023). Whether these ESPCs retain the hematopoietic stem-progenitor cells’ (HSPC) surface marker antigen CD34 (CD133^+^CD34^+^), or negative for CD34 (CD133^+^CD34^−^), these progenitor stem cells co-express the ACE2 cell-surface antigen, the latter being the primary receptor for SARS-CoV-2 entry into the ACE2^+^(CD133^+^CD34^+/−^) ESPC. It is perceived that this virus prefers the co-expression of CD133 with ACE2 for a better and more direct anchorage onto the ACE2^+^ ESPC and subsequently recruits, activates, and impairs the T-lymphocytes.

In contrast, in HIV-1 infection, the CD34^+^ HSPC also become susceptible to this virus but in an indirect manner following the infection of CD4^+^ T-lymphocytes, as we had reported that the HIV-1 induced inhibition of differentiation of this HIV-1 infection-resistant CD34^+^ HSPC to be an indirect phenomenon (Koka et al., 1998; Koka et al., 1999).

### Both T and B lymphocytes lack the ACE2 receptor

Expression of the SARS-CoV-2 receptor ACE2 is lacking in the immune response T- and B-lymphocytes (Hikmet et al., 2020). However, these immune cells become pathogenic to this virus, eliciting a profuse cytokine storm through the cytokine dysregulation in these cells; interestingly, this is in an indirect manner following infection of the CD133^+^CD34^+/−^ ESPC through this virus’ primary receptor ACE2 on the cell surface (Fajgenbaum and June 2020; Gheblaw et al., 2020; Koka et al., 2020; Liu et al., 2021; Kotani et al., 2022).

### Mechanistic consequences of HIV-1 infection of CD4^+^ T-lymphocytes on HSPC

**FIGURE 1:**
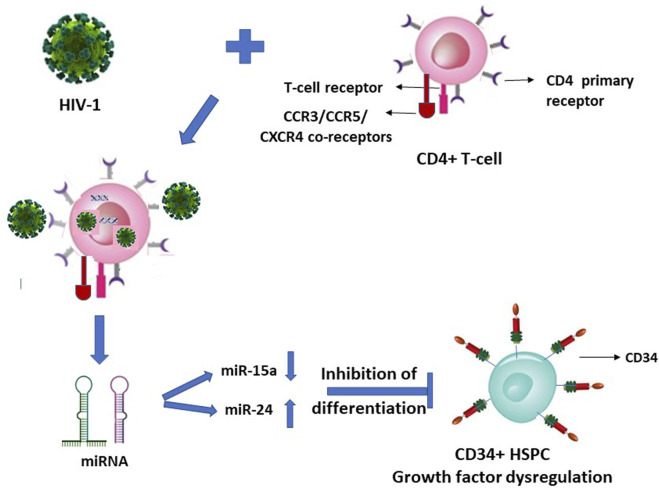
HIV-1 infection of CD4^+^ T-lymphocytes indirectly inhibits the differentiation of this virus infection-resistant (primary CD4 receptor-lacking) CD34^+^ hematopoietic stem-progenitor cells (HSPC).

We reported that upon HIV-1 infection of the CD4^+^ T-lymphocytes, there occurs a secretion of specific microRNAs from these infected cells in a differential manner with miR-15a and miR-24 regulated down and up, respectively (Figure 1) (Padmanabhan et al., 2020). When the CD34^+^ HSPC were exposed to these cells’ (CD4^+^)-free and virus (HIV-1)-free supernatants comprising these differentially expressed miRNAs, the *in vitro* assayed CFU-GM and BFU-E colony forming activity (CFA) of the purified cell-/virus-free supernatant-exposed CD34^+^ HSPC was decreased or inhibited, viz. hematopoiesis (CFA) (Padmanabhan et al., 2020).

### Potential mechanistic consequences of SARS-CoV-2 infection of ACE2^+^ CD133^+^ ESPC

**FIGURE 2:**
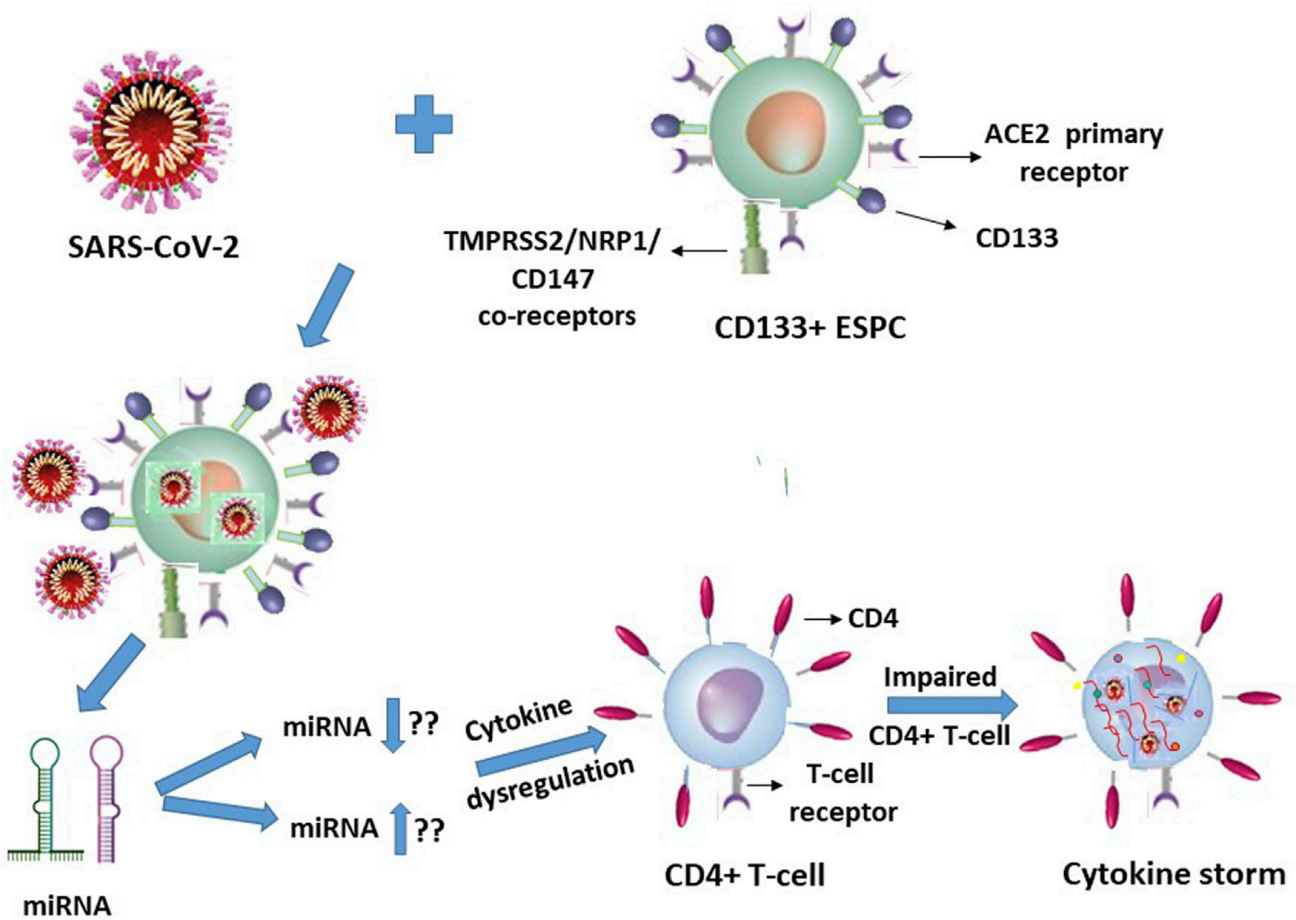
SARS-CoV-2 infection of CD133^+^ACE2^+^ endothelial stem-progenitor cells (ESPC) indirectly causes cytokine storms in this virus infection-resistant (primary ACE2 receptor-lacking) CD4^+^ T-lymphocytes.

In view of our earlier report on HIV-1 (Padmanabhan et al., 2020), it is quite logical and may even be plausible that specific but hitherto unknown putative miRNA molecules will be secreted or released by the SARS-CoV-2 infected ACE2^+^CD133^+^CD34^+/−^ ESPC, which in turn may interact with the T-lymphocytes in an indirect manner to trigger cytokine storms of these immune response cells (Figure 2).

### Multi-faceted growth factor or cytokine regulation mechanisms and convergence on their alternate reciprocal progenitor stem cell pathogenic dysregulation events in these virus infections

It is suggestive from Figures 1, 2 (with the latter as we have projected in this article), that expression of the cellular growth factors or cytokines that sustain the cellular functions and their survival are impeded or disrupted in these infections by HIV-1 and SARS-CoV-2. The modes of pathogeneses are different even though the viruses converge on the cellular dysregulatory objectives through these two different pathogens. Both the precursor stem-progenitor cells and their terminally differentiated lineages into the immune regulatory cells are targeted by these viruses, although through mechanistic routes that are divergent but may be perceived as reciprocally targeted mechanistic routes.

## Discussion

The “recruitment” or more appropriately, involvement of different stem-progenitor cell types, precursor potencies (viz. pluripotent, multipotent), or differentiation pathways, to cause pathogenicity, either directly or indirectly by these two viruses upon infection of the respective target cell populations, suggests or denotes the spectre of stem cells being targeted by different viruses upon infection.

The primary target cells of these two viruses possess the receptors for direct virus entry but the secondary targeted cells may not need or lack the receptors for the virus induced pathogenesis. In such instances, the viruses may utilize the noncoding long RNA (nclRNA) or microRNA (miRNA) molecules of the initial or primary host cellular targets, to reach out to the secondary host cells in the cellular microenvironments. Subsequently, the normal miRNA regulation is perturbed and potentially specific miRNAs are secreted more likely in a differential manner. In turn, the putative dysregulated miRNAs interact with the secondary infection-resistant cells (of each virus) through intra-cytoplasmic interactions with specific messenger RNA (mRNA) targets causing post-transcriptional dysregulation that regulate growth factor cytokines.

With respect to the stem-progenitor cell pathogenesis, whereas HIV-1 causes secondary damage to the CD34^+^ HSPC via miRNA of the CD4^+^ T-lymphocytes, SARS-CoV-2 inflicts its invasion first upon the CD133^+^ ESPC and the released “putative” miRNAs secondarily target the CD4^+^ T-lymphocytes. We postulate that in SARS-CoV-2 infection the infected CD133^+^ cells cause secondary influence via miRNAs that act on the T- and B- lymphocytes (Figure 2).

This triggers a spate of multiple cytokines to be released from the ESPC-secreted miRNA-influenced T-lymphocytes that are undesirable cytokine storms especially in vulnerable comorbidity-diagnosed individuals, instead of maintaining their normal cytokine homeostasis levels and also rather than controlled efficacy producing anti-virus levels. The latter may transiently be controlled upon therapeutic interventions or by the immune cells (of non-compromised/non-vulnerable individuals) in circulation in the human body. Thus, the most interesting feature of these two viruses induced cellular damages is that the stem-progenitor cells are being targeted either directly or indirectly. In addition, the noncoding miRNAs may play a significant role in triggering the cytokine storm generation of the T-lymphocytes, and in B-lymphocytes through antibody responses, despite these T- and B-cells lacking the host cell receptor ACE2 to facilitate a direct entry of SARS-CoV-2.

Interstitial seepage of the virus particles—including their sub-genomic, or sub-transcriptomic fragments, or viral peptides or proteins—into other primary receptor-lacking productive infection-resistant cells, may occur once such nonspecific “tertiary” presence or direct virus-receptor interaction-unrelated cell membranes become porous or permeable, due to infection of the primary target cell. This may occur since the presence of virus in the cellular microenvironments or in systemic circulation may cause a minimal random presence of virus-related molecular entities in different cells. A purported latency of these viruses either in the hemangioblasts or in the T-lymphocytes needs to be explained as to how HIV-1 or SARS-CoV-2 respectively can be elusive in these cells to the antiviral therapies at a time when these respective viruses are susceptible to the antiviral treatments in the cells of their productive primary target cell infections. Such therapeutic efficacy occurs even to the extent of reducing the viral loads to undetectable levels (<50 copies/mL HIV), not only in these primary target cells expressing the viruses’ receptors + coreceptors but also in the infected human sera. It may be possible that virus containment by antivirals can occur to such an extent that nonspecific virus presence due to any interstitial or other similar modes of seepage into the infection-resistant cells delays their otherwise imminent apoptotic cell death due to virus entry/presence even prior to replication or productive infection. Naturally, this leads to the conclusion that the current antiviral treatments fall short of a complete 100% efficacy. However, a nearly undetectable ART efficacy to contain the systemic HIV levels may be similarly and clinically applicable to other cells in parallel with such minimal virus levels presence due to nonspecific entry (seepage) into cells through certain nonspecific receptors. These ligands in particular are such as those involved in the context of adhesion molecules expressed on CD133^+/−^ (CXCR4^-^) medullablastoma cancer stem cells (HTB-186 that expressed ICAM-1, VCAM-1, LFA-3), susceptible to natural killer (NK) cell mediated activity-killing (Castriconi et al., 2007).

Such LFA-1/ICAM (CD11a/CD18) mediated HIV entry into the T-lymphocytes apart from the virus’ natural CD4 receptor also when co-expressed on the same Jurkat cells was reported (Hioe et al., 2001). It therefore may not be considered as a primary infection, nor a mechanism for latency of the virus, possibly allowing seepage of the virus into T-cells that are affected dually from and due to the primary infection of the CD4^+^ cells. Hence, HIV presence considered as latent reservoirs even in the T-lymphocytes, or CD133^+^ stem-progenitor cells, may not hold ground since their virus loads in the secondary non-tropic CD133^+^ cell presence due to nonspecific seepage, as also facilitated by integrins such as LFA-1, are presumably at similar levels and in parallel to the virus’ primary target cells while under ART. If the CD133^+/−^CD34^+/−^ cells are treated with any growth factors, as also reported (McNamara et al., 2013), then the primitive phenotypic cells are differentiated into mature phenotypes with and acquirement of T-lymphocyte lineage markers, thus paving the way for the resumption of virus infection. In such an instance, the presumed latent reservoir cells in fact would have acquired the mature T-lymphocyte phenotypes permissive for productive virus infection. The virus entry solely through the secondary receptors such as CXCR4 and non-utilization of the primary receptors is at most a nonproductive virus entry or seepage which are then presumably “disabled” viruses due to intracellular growth factor dysregulation (Figure 1) and consequently results in suspension or halt of apoptosis *in vivo*. This occurs when virus loads are reduced to undetectable levels across the productive and nonproductive primary and secondary cells respectively, by the antiretroviral treatments (McNamara et al., 2013). A resumption of apoptosis of the secondary cells may occur if the ART is ceased or the growth factors are exogenously replenished. However, functional resumption of such imminent or instant apoptosis as an innate immune response would prevent the renewed spread of virus particles to the surviving T-lymphocytes *in vivo* when what may be the so-called ‘dormant’ virus particles are reactivated either by a cessation of ART or through a resumption of supply of the growth factor cytokines triggered by intercellular miRNA involvement (Figure 1).

A similar type of argument may well be advanced and applicable to SARS-CoV-2 entering their primary infection-resistant ACE2-negative target cells, using manipulated Jurkat cells *in vitro,* reportedly via LFA-1, similar to HIV-1 vis-à-vis LFA-1 (Hioe et al., 2001; Shen et al., 2022). Herein lies a question if it is the LFA-1 that possesses the dubious distinction of participating in the promiscuity of providing a spurious correlation, or genuine refuge, to multiple viruses. The reported coincidental occurrence or involvement of such integrins (adhesion molecules) implicated in the presence of HIV-1 and SARS-CoV-2 (Hioe et al., 2001; Castriconi et al., 2007; McNamara et al., 2013; Shen et al., 2022), in the respective infection-resistant cells **(** Figures 1, 2 **)**, raises doubts on the premise of latent reservoir cells of viruses.

The cellular damage from the early onset of, for example, a cytokine storm in the secondary target T-lymphocytes due to SARS-CoV-2 primary infection of the ACE2^+^CD133^+^ ESPC may render the indirectly influenced T-cells’ surface become susceptible to diffusion of multiple molecular entities including that of the virus **(** Figure 2 **)**. These may be mechanistically inconsequential to an inducement of potential CD133^+^ ESPC-secreted putative miRNA or other such entity-driven inflammatory cytokine storms of the T-lymphocytes. Logically, the virus is expected to be abundant in the infected dually positive ACE2^+^ (CD133^+^ ESPC) cells. If the virus presence in T-cells is possible then that may be expected possibly due to a nonspecific interstitial seepage into, or due to an interaction nonspecific in nature, with the antigens of the circulating T-cells. We know that despite the virus causing T-cell cytokine storms in infected patients, some of them survive and some die. Hence, it is highly unlikely that ACE2-negative T-cells can compete with ACE2^+^ (hemangioblasts, CD133^+^CD34^+/−^) (Sidney et al., 2014; Koka et al., 2020; Hassanpour et al., 2023) cells in receiving or harbouring the virus to such an extent that can cause the death of SARS-CoV-2 infected patients unless as an indirect consequence on the secondary T-cells.

## Conclusion and future directions

Hence, we postulate that putative miRNA molecules may be involved in the SARS-CoV-2-induced pathogenesis of the CD133^+^ endothelial stem-progenitor cells in an epigenetic regulatory mechanism of the miRNA-targeted messenger RNAs (mRNA) (Koka et al., 2020; Padmanabhan et al., 2020; Koka and Reddy, 2023) of the T- or B-lymphocytes. These cells, in turn, produce not only excessively impaired immune responses but also cause potentially lethal or long-lasting heart and lung damage in virus-infected individuals or patients. Therefore, it will be very important to know what, if any, putative miRNAs and the levels of which are released by the SARS-CoV-2-infected CD133^+^ ESPC that interact with the mRNA of the cytokines expressed by the CD4^+^ T-lymphocytes. Thus, miRNAs as therapeutic efficacy to correct their own deleterious dysregulation as well as that of its target mRNA of the interacting cells may be investigated for treatments to contain cytokine storms of T-lymphocytes, or abnormal B-cell humoral antibody responses (and also of cytokines such as IL-10 in relatively low instances), in SARS-CoV-2 infection. Finally, virus-entry inhibitory therapies (Higashi-Kuwata et al., 2023) besides the vaccines may serve to further contain the deleterious effects of SARS-CoV-2 in conjunction with the post-virus-entry released cellular microRNAs to be targeted by drug candidates. These may also include the very same secreted and transported miRNAs and with their levels to be regulated, that will be useful for interventional treatment efficacies for COVID-19.

## Data Availability

The original contributions presented in the study are included in the article/supplementary material, further inquiries can be directed to the corresponding author.
